# Chaiyu-Dixian Formula Exerts Protective Effects on Ovarian Follicular Abnormal Development in Chronic Unpredictable Mild Stress (CUMS) Rat Model

**DOI:** 10.3389/fphar.2020.00245

**Published:** 2020-03-24

**Authors:** Hui-Xian Xu, Shu-Xia Lin, Yuewen Gong, Zi-Xuan Huo, Cheng-Yun Zhao, Hong-Mei Zhu, Sheng-Yan Xi

**Affiliations:** ^1^Cancer Research Center, School of Medicine, Xiamen University, Xiamen, China; ^2^Department of Traditional Chinese Medicine, School of Medicine, Xiamen University, Xiamen, China; ^3^College of Pharmacy, Rady Faculty of Health Sciences, University of Manitoba, Winnipeg, MB, Canada

**Keywords:** Chaiyu-Dixian formula, chronic stress, follicle development, PI3K/Akt, brain-derived neurotrophic factor, ovary

## Abstract

**Background:**

Chronic stress has been known to impair the female reproductive function, but the mechanism remains to be further investigated. Chaiyu-Dixian Formula (CYDXF) has been reported to regulate human endocrine disorders clinically. However, whether this formula can affect chronic stress-induced ovarian follicular development is not clear.

**Aim of the study:**

To examine effects of CYDXF on follicular development and explore possible mech anisms in a chronic unpredictable mild stress (CUMS) model.

**Materials and Methods:**

Adult female rats were randomly divided into 5 groups control group, CUMS group (saline treatment), CUMS+Estradiol (E_2_) (0.1 mg/kg) group, CUMS+CYDXF (2.73 g/kg) group, and CUMS+CYDXF (5.46 g/kg) group. Body weights and behavioral tests were documented. Serum hormone levels were determined by enzyme-linked immunosorbent assay (ELISA). Western blotting was used to detect the protein levels in the PI3K/Akt pathway and brain-derived neurotrophic factor (BDNF). The follicles were analyzed and classified according to their morphological characterization.

**Results:**

CYDXF relieved depression-like behaviors and ameliorated the abnormality in rat estrous cycle within the rat model of CUMS. Moreover, CYDXF could regulate endocrine disorders, increase the proportion of antral follicles as well as decrease the proportion of follicular atresia, which suggested that CYDXF could alleviate abnormal follicular development and improve overall ovarian function. Furthermore, CYDXF also activated the BDNF-mediated PI3K/Akt signaling pathway.

**Conclusions:**

CYDXF (at dose of both 2.73 and 5.46 g/kg) attenuated chronic stress-induced abnormal ovarian follicular development by relieving depression-like behaviors and improving ovarian function through partly the regulation of the BDNF-mediated PI3K/Akt pathway.

## Introduction

Chronic stress can affect the body's physiological and mental health, including its metabolic and immune systems. It is one of the risk factors for anxiety, depression, cardiovascular disease, and other diseases (McEwen, 2008; Karisetty et al., 2017). Due to anatomical and physiological structures, women in the reproductive age are more susceptible than men to developing stress-related psychiatric disorders such as depression and anxiety. The prevalence of psychiatric disorders in women is approximately 2 to 3 times higher than in men (Westenbroek et al., 2004; Ter Horst et al., 2009). Some important relationships between sex hormones and depression have been observed (Peltier et al., 2019). Altered reproductive hormone levels have been associated with the pathophysiology of depressive disorders (Smeeth et al., 2019). Moreover, chronic stress has certain impacts on female reproductive functions, which can lead to disorders of ovulation and infertility (Zangeneh and Shooshtary, 2009). For example, repeated restrained stress (RRS) can damage oocyte development (Gao et al., 2016); heat stress can reduce estradiol levels produced by cumulus-oocyte complexes (COCs) (Paes et al., 2016); chronic cold stress may change follicular development, leading to a reduction of antral follicles via an intraovarian sympathetic activation (Dorfman et al., 2003); chronic unpredictable mild stress (CUMS) not only inhibits the development of secondary follicles and antral follicles in mice, but also increases the atresia of follicles (Wu et al., 2012a).

Follicles are the basic units of the ovary. They can produce eggs as well as secrete steroid hormones. In mammalian ovaries, primordial follicles develop during the fetuses (such as primates) or develop early in newborns (such as rodents) (Hirshfield, 1991; Fortune, 1994). The follicles in the primordial follicle pool undergo a series of recruitment, selection, and dominance, eventually resulting in ovulation or atresia. Therefore, maintaining the growth and development of follicles plays an important role in women's reproduction. This process is not only affected by different hormones and growth factors through endocrine, autocrine, and paracrine patterns, but also regulated by a variety of signaling pathways (Wu et al., 2012b; Fernandois et al., 2016; Hayes et al., 2016; Wen et al., 2018). The brain-derived neurotrophic factor (BDNF) is one of these factors. Although BDNF is primarily produced by the brain and has an influence on the growth and survival of neurons, it may be associated with depression (Rasmussen et al., 2009; Larsen et al., 2010). Moreover, BDNF is also expressed in the ovaries (Bodis et al., 2014). After binding to its specific receptor, BDNF activates its downstream phosphoinositide-3 kinase-protein kinase B-mammalian target of rapamycin (PI3K-AKT-mTOR) signaling pathway (Martinowich and Lu, 2008). The PI3K-AKT-mTOR signaling pathway plays a crucial role in cell proliferation, differentiation and apoptosis (Chen et al., 2016), and is involved in the activation of primordial follicles and the meiosis of oocytes (Zheng et al., 2012). Moreover, the BDNF-activated PI3K-AKT-mTOR signaling pathway is reported to be essential in female reproductive diseases such as polycystic and premature ovarian failure, which may be induced by chronic unpredictable mild stress (Divyashree and Yajurvedi, 2016; Sun et al., 2017; Fu et al., 2018).

In recent years, the potential value of traditional Chinese medicine (TCM) has been recognized because TCM exerts profound activity in many diseases and has relatively few side effects (Efferth et al., 2007; Sun et al., 2017). Chaiyu-Dixian Formula (CYDXF) is one of the traditional Chinese empirical formulas, which consists of Radix Bupleuri, Radix Curcumae, Radix Rehmanniae Praeparata, Epimedium Herb, Rhizoma Pinellinae Praeparata, Poria, Radix Codonopsis, Radix Salviae Miltiorrhizae, and Glycyrrhizae. Previous studies showed that CYDXF could regulate many disorders of the endocrine system and had an antidepressant effect (Li and Zhu, 2016; Zhou et al., 2017). Pharmacological studies have shown that major bioactive substances isolated from CYDXF possess activities against gynaecological disorders and can also treat depressive disorders (Papoutsi et al., 2006; Yang et al., 2011; Gong et al., 2016; Yan et al., 2018; Liu et al., 2019). However, the role of CYDXF in chronic stress-induced follicular abnormal development has not been examined.

Our previous study found that the hippocampal BDNF expression was significantly decreased in chronic stress-induced depressive rats (Zhou et al., 2018). However, the effects of ovarian BDNF in chronic stress-induced rats still needs further investigation. At the same time, whether the PI3K-AKT-mTOR signaling pathway is involved in chronic stress-induced follicular development in ovaries needs to be further elucidated. Therefore, this study aims to explore the biological activities and mechanism of CYDXF in a rat model of chronic stress-induced ovarian follicular development.

## Materials and Methods

### Experimental Animals

Thirty-five adult female Sprague-Dawley rats, weighing 190-200 g and aged 7-8 weeks, were purchased from the SLAC Laboratory Animal Co. Ltd in Shanghai, China [License No. SCXK (Hu) 2016-0003]. Animals were allowed to adapt for seven days before the experimental procedures, and rats with an abnormal estrous cycle were discarded. The remaining rats were housed in polypropylene cages under controlled conditions at 22-24 °C and photoperiod of lights on 07:00 a.m.-19:00 p.m. with free access to food and tap water unless food or water deprivation were needed. They were randomly assigned to 5 groups: control group, CUMS group (0.9% normal saline, 10 mg/kg), CUMS+Estradiol (0.0001 g/kg) group, CUMS+CYDXF (2.73 g/kg) group, and CUMS+CYDXF (5.46 g/kg) group. The control animals were housed separately and had no contact with the stressed groups. Stressed and control animals were kept in separate rooms of the Animal Center of Xiamen University [No. SYXK (Min) 2018-0009]. The body weight of rats was recorded once every week. All protocols in this animal experiment were conducted in strict accordance with the animal care and use guidelines of the Ministry of Science and Technology of the People's Republic of China, and the study was approved by the Laboratory Animal Administration and Ethics Committee of Xiamen University.

### Stress Model

The stressed groups were individually housed and received a 28-day stress procedure according to previous studies, with minor modifications (Willner, 1997). They were administered every day between 9:00 a.m. and 11:00 a.m., excluding the 24 h duration stressors. Stress procedures included: (1) 24-h food deprivation, (2) 24-h water deprivation, (3) 24-h cage tilt (45 degrees from the horizontal), (4) 24-h wet cages, (5) overnight illumination, (6) 2-h physical restraint, (7) 5-min cold swim at 4°C, after which they were toweled dry, (8) 5-min shaker stress, (9) 24-h placement in an empty cage and (10) 1-min tail pinch (1 cm from the end of the tail). Habituation can develop when animals are repeatedly exposed to a predictable stressor—such as the chronic restraint stress (CRS) model. Thus, all stressors were randomly scheduled over a 1-week period and repeated throughout the 4-week experiment (**Table 1**) to ensure the unpredictability of the stressors.

**Table 1:** Schedule and procedure of stressors used during 28 days of CUMS.

Day	Stressor	Duration
Monday Tuesday Wednesday Thursday Friday Saturday Sunday	Physical restraint Overnight illumination Placement in an empty cage Cold swim at 4°C Tail pinch Shaker stress Food deprivation Water deprivation Cage tilt (45°) Overnight illumination Wet cages Placement in an empty cage Food deprivation Water deprivation	2 h 12 h 24 h 5 min 1 min 5 min 24 h 24 h 24 h 12 h 24 h 24 h 24 h 24 h

### Experimental Drugs

CYDXF consists of Chai Hu (Radix Bupleuri) (8.85%), Yu Jin (Radix Curcumae) (8.85%), Shu Di Huang (Radix Rehmanniae Praeparata) (13.27%), Xian Ling Pi (Epimedium Herb) (13.27%), Fa Ban Xia (Rhizoma Pinellinae Praeparata) (10.62%), Fu Ling (Poria) (13.27%), Dang Shen (Radix Codonopsis) (13.27%), Dan Shen (Radix Salviae Miltiorrhizae) (13.27%) and Gan Cao (Glycyrrhizae) (5.31%) (**Table 2**). The major bioactive components in CYDXF are saikosaponin D (Li et al., 2017), curcumin (Bamba et al., 2011), iridoids glycosides (Liu et al., 2012), icariin (Xue et al., 2016), pinellic acid (Nagai et al., 2002), the triterpene fraction and the polysaccharide fraction (Rios, 2011), polysaccharides and saponins (Li et al., 2009), tanshinone and cryptotanshinone (Chang et al., 1990) as well as liquiritigenin (Kim et al., 2004). Each herb in CYDXF was purchased from the Xiamen Yanlaifu Pharmaceutical Co., Ltd. (Xiamen, China) and identified by Professor Yingkun Qiu (School of Pharmaceutical Sciences of Xiamen University, China). All voucher specimens were deposited at the Xiamen Botanical Garden (Herbarium Code: XMBG) for future reference. Estradiol valerate (E_2_) was produced by Bayer Healthcare Co., Ltd. Guangzhou Branch, with a product lot number of J20171038.

**Table 2:** Contents of Chaiyu-Dixian Formula (CYDXF).

Chinese name	Common name	Botanical name	Weight	Voucher numbers	Part used
Chai Hu	Radix Bupleuri	*Bupleurum chinensis* DC.	10 g	181201902	Root
Yu Jin	Radix Curcumae	*Curcuma aromatica* Salisb.	10 g	180501	Tuber
Shu Di Huang	Radix Rehmanniae Praeparata	*Rehmannia glutinosa* (Gaertn.) DC.	15 g	181201	Rhizome
Xian Ling Pi	Epimedium Herb	*Epimedium brevicornu* Maxim.	15 g	181101904	Aerial part
Fa Ban Xia	Rhizoma Pinellinae Praeparata	*Pinellia ternata* (Thunb.) Breit.	12 g	180701	Tuber
Fu Ling	Poria	*Poria cocos* (Schw.) Wolf.	15 g	190428	Sclerotium
Dang Shen	Radix Codonopsis	*Codonopsis pilosula* (Franch.) Nannf.	15 g	190620	Root
Dan Shen	Radix Salviae Miltiorrhizae	*Salvia miltiorrhiza* Bunge	15 g	180901	Root & Rhizome
Gan Cao	Glycyrrhizae	*Glycyrrhiza uralensis* Fisch.	6 g	181001	Rhizome

### Main Reagents and Kits

In the study, the following materials of rabbit polyclonal anti-BDNF antibody were purchased from Proteintech Group, Inc. (Chicago, USA): product lot No. Cat#28205-1-AP, rabbit polyclonal anti-PI3K p110 (beat) antibody, product lot No. Cat#20584-1-AP, rabbit polyclonal anti-AKT antibody, product lot No. Cat#10176-2-AP, and rabbit polyclonal anti-mTOR antibody, product lot No. Cat#20657-1-AP. Rabbit polyclonal anti-p-AKT antibody, product lot No. 4060S and rabbit polyclonal anti-p-mTOR antibody, product lot No. 5536S, were purchased from Cell Signaling Technology, Inc. (Danvers, USA). MLGR-E30267 Rat Estradiol (E_2_) ELISA Kit, product lot No.ml002871, MLGR-E30274 Rat Follicle-stimulating hormone (FSH) ELISA Kit, product lot No.ml002872, MLGR-E30447 Rat Luteinizing Hormone (LH) ELISA Kit, product lot No.ml002860, and MLGR-E31019 Rat Progesterone (Prog) ELISA Kit, product lot No.ml002894, were produced by Shanghai Enzyme-linked Biotechnology Co., Ltd. (Shanghai, China).

### Medicinal Preparation and Administration

Based on the clinical dosage, CYDXF contains 113g crude herbs, which is equivalent to a dosage of 11.6 g (dried medicinal herb)/kg for rats, according to the formula that converts equivalent drug dosages based on body surface areas from human to rats [Human equivalent dose (HED) = Animal dose (mg/kg) *multiplied by* $\frac{\text{Animal }Km}{\text{Human }Km}$ (Km factor of a 60 kg adult is 37 and Km factor of a 0.15 kg rat is 6) ] (Reagan-Shaw et al., 2008). To prepare this formula, a total of 113g of mixed CYDXF crude herbs were immersed in 1130 mL of distilled water for 30 min and boiled for 1 h to yield a final volume of 150 mL. The decoction was filtered with 8 layers of surgical gauze. Herb residues were again soaked in 600 mL water, boiled for 1 h, and filtered again. Both filtered decoctions were combined and concentrated with rotary evaporation (Shanghai Yarong Biochemistry Instrument Factory, Shanghai, China) at 58^0^C until a final volume of 90 mL was reached, then lyophilized with freeze dryer (Beijing Songyuan Huaxing Technology Development Co., Ltd., Beijing, China) to get the extract. The weight of the final freeze-dried powder of each dose of CYDXF (113g) was 26.58g. The extraction yield was 23.52% and the concentration of the final filtered decoction was 0.30 g/mL. The lyophilized powder was stored at 4°C until use. The dosages of 2.73 and 5.46 g /kg [equal to 11.6 and 23.2 g (dried medicinal herb)/kg, respectively] of Chaiyu-Dixian Formula (CYDXF) were chosen, which were equivalent to 10 and 20 times of the clinical recommended human daily dose, respectively. The powder was reconstituted with distilled water to 0.27 g/mL, 0.55 g/mL respectively. The model group received normal saline (0.9%, 10 mL/kg). E_2_ was dissolved in distilled water to a concentration of 0.01 mg/mL. All medicines were administered to rats by gavage in a volume of 10 mL/kg once per day.

### Estrous Cycle Determination

The vaginal smears were performed using methylene blue staining at 11:00 a.m. for ten consecutive days, beginning with the 18^th^ day of the CUMS procedure, and were observed under a light microscope (Marcondes et al., 2002). Briefly, there were three types of exfoliated cells that could be observed: the oval nuclear epithelial cells (NE), the irregular-shaped anucleated epithelial keratinocyte cells (EK), and the little round leukocyte cells (L). The four phases of the estrous cycle were determined by the proportion among them. In proestrus, nuclear epithelial cells were mainly observed, occasionally with a few of epithelial keratinocyte cells; in estrus, the vaginal smear primarily consists of cornified cells with no nucleus and a small number of nucleated epithelial cells. When the smear consisted of the same amount of leukocyte, cornified and nucleated epithelial cells, it was considered a metestrus phase; in diestrus, a predominance of leukocytes and a small portion of nuclear epithelial cells were detected.

### Behavioral Tests

Animals were placed in the testing room and acclimated for 0.5 h before the test. All tests were conducted between 7:30 a.m. and 11:30 a.m. in a dim and quiet room. After being tested, rats were returned to the holding rooms.

Open Field Test (OFT): The open field test was aimed to evaluate the rodents' spontaneous spatial exploration activity (Jindal et al., 2013). Briefly, the apparatus consisted of a square arena (100 cm × 100 cm × 50 cm) with a black floor and a four-sided black wall. Each rat was placed gently on the center of the area and were allowed to explore freely for 5 min during the test. The activity of rodents, including central and peripheral activity, total movement distance, and times of rearing and grooming behaviors, were recorded by a video camera connected to a computer 1.5 m above the center of the floor. After each test, the apparatus was cleaned with a solution of 75% ethanol and was dried.

Forced Swim Test (FST): The FST was performed to assess the depression-like behavior of rats (Walf et al., 2009). Rats were individually placed in a transparent plexiglass cylindrical container (30 cm in diameter, 80 cm in height) with water at 25°C and filled to 50 cm of depth and videotaped for 5 min. In this container, rats cannot reach the bottom or escape. After the test, total immobility time was analyzed by two independent observers to avoid bias, and average time was recorded. Contrary to activity (swimming or diving behavior), immobility was defined as passive, floating-like behavior lasting longer than 1 second. After each test session, the water was changed and containers were cleaned thoroughly in order to eliminate odor. Rats were dried and returned to their home cages.

Sucrose Preference Test (SPT): The sucrose preference test was used as an index of stress-induced anhedonia in rodents (Patki et al., 2013). Briefly, rats were kept separately in cages and trained to consume from two identical bottles. On the first day of SPT, two bottles filled with 100 mL of 1% sucrose solution were given to rats. On the 2^nd^ day, one bottle was filled with 100 mL of 1% sucrose solution and the other 100 mL of distilled water. In the meantime, the bottles were interchanged after 12 h. On the third day, rats were deprived of food and water. The test was conducted on the fourth day. Two bottles of water and sucrose were given to rats for 24 h, and the placement of them was interchanged after 12 h. The weight of the bottles was measured before and after the test. The sucrose preference was calculated by the following formula: Sucrose preference = sucrose consumption/ [total consumption (sucrose + water)]×100%.

Elevated Plus Maze (EPM): The elevated plus maze was to assess anxiety-like behavior in rats (Aldridge et al., 2005). The apparatus was 50 cm above the floor and consisted of 4 arms (45 cm long, 10 cm wide), of which two were open and two were closed. Four arms extended from a central platform (10 cm× 10 cm) shaping like a plus sign. Each rat was placed gently in the central platform facing the open arms to crawl freely for 5 min. Percentage of time spent in open and closed arms, number of entries into open arms (with all four paws), along with the head dipping and rearing of each rat were recorded. After each trial, the apparatus was wiped down with 75% ethanol.

### Biochemical Analysis

Biochemical analysis was conducted after all behavioral tests were finished. Rats were anesthetized and sacrificed on the 30^th^ day. Serum hormone levels and ovarian protein expression were assayed. The ovarian morphological and histological characteristics were observed.

#### Sample Collection

Peripheral blood was collected and its serum was obtained by centrifugation of blood at a speed of 3000 r/min for 15 min. After that, the supernatant was separated and stored at -80°C until use. For morphological evaluation, the right ovaries were removed and fixed in 4% paraformaldehyde at -20°C for at least 24 h, then dehydrated, embedded in paraffin, serially sectioned (with a thickness of 3 μm), and stained by hematoxylin-eosin. For protein level measurement, the left ovaries were quickly removed, dipped in liquid nitrogen, and stored at -80 °C.

#### ELISA Analysis

We used the enzyme-linked immunosorbent assay to detect the hormone levels in serum. The contents of follicle-stimulating hormone (FSH), estradiol (E_2_), luteinizing hormone (LH), and progesterone (Prog) in serum were determined by commercially available ELISA kits, according to the manufacturer's instructions.

### Western Blot Analysis

Total protein levels of PI3K, AKT, mTOR, and BDNF were determined by western blotting in the left ovaries. The proteins were extracted by homogenizing the ovaries in radio immunoprecipitation assay (RIPA) lysis buffer (Beyotime, Nanjing, China) and centrifuged at 12000 rpm for 5 min. The supernatant was obtained and the protein concentration of each sample was assessed using a bicinchoninic acid (BCA) protein assay kit (Beyotime, Nanjing, China). The samples containing equal amounts of total proteins were loaded in each track and separated using 10% sodium dodecyl sulfate-polyacrylamide gel electrophoresis (SDS-PAGE), and subsequently electro-transferred to polyvinylidene fluoride (PVDF) membranes. After treating with blocking solution (Tris-buffered saline Tween containing 5% skim dry milk), the membranes were incubated overnight at 4 °C with specific primary antibodies. After this process, the membranes were rinsed with TBST and incubated with HRP-conjugated secondary antibody for 1 h at room temperature. After washing with TBST three times, the proteins of the bands were detected by the enhanced chemiluminescence (ECL) detection reagent (Beyotime, Nanjing, China). The optical density of protein bands on the membranes was analyzed by the ONE-DScan program system.

### Ovarian Histological Evaluation

The right ovarian tissues were excised, rinsed and fixed in 4% paraformaldehyde. After 12 h fixation, the specimens were embedded in paraffin and serially sectioned (3 μm), mounted on glass slides, and stained with hematoxylin and eosin (HE). The presence of the primordial, primary, secondary, antral, and atretic follicles as well as corpus luteum were analyzed and classified according to their morphological characterization (Osman, 1985; Myers et al., 2004; Yoshida et al., 2009). The follicles were counted in every 10^th^ section of the whole ovaries by two independent researchers.

Briefly, follicle stages were classified under the following definitions. Primordial follicles showed a partial or complete layer of flattened pregranulosa cells surrounding an oocyte. Primary follicles were defined as an oocyte that was encircled by a single layer of columnar or cubic granulosa cells (GCs). Secondary follicles appeared on two or more layers of granulosa cells with no cavity and antral follicles had formed a cavity filled with fluid—the antrum. The antral follicle was counted when nuclei was observed. Follicles were judged as artresia when showing deformation and/or had pyknotic oocyte nuclei, as well as granulosa cells showing shrinkage or fragmentation. Corpus luteum contained luteal cells which had eosinophilic cytoplasm and basophilic nuclei.

### UHPLC/ESI-MS Analysis

The major chemical components of CYDXF decoction were examined using ultra-high performance liquid chromatography (UHPLC) equipped with an electrospray ionization mass (ESI-MS) detector. Analysis on HPLC was performed on a Cosmosil 5C18-MS-II column (250 × 4.6 mm i.d., 5 μm, Nakalai Tesque Co. Ltd., Kyoto Japan) with mobile phase consisting of water (A) and acetonitrile (B) in a gradient program: A from 95% to 30% and B from 5% to 70% during 0-35 min, A from 30% to 0% and B from 70% to 100% during 30-31 min. Then A were kept 0% at 31.1-40 min, and lastly, A and B ran back to 95% A and 5% B when 40.1 min and kept there during 40.1-45 min, at a flow rate of 1 mL/min. The column was maintained at room temperature and the injected volume was 5 μL. The mass spectrometer was equipped with a high resolution ESI detector and were used to record the HPLC chromatograms. The MS spectra were recorded on a Thermo Q-Exactive system. The positive and negative electrospray voltages were 3.5 Kv and 2.5 Kv, the capillary temperature was set at 300 °C, and the mass range was 100-1000. 

### Statistical Analysis

All results were presented as mean ± standard mean of error (SEM) ($\bar{x}$± sem). Data were analyzed by SPSS 21.0. Normal distribution and homogeneity test of variance between groups were performed. One-way analyses of variance (One-Way ANOVA) was used to compare the means of multiple groups. The least significant difference (LSD) method was selected for post hoc analysis. If there was one inconsistency, the Kruskal-Wallis H test of multiple independent samples in nonparametric statistics was used for statistical processing. GraphPad Prism Version 7 software (GraphPad Software, USA) was used for drawing statistical graphs. A value of *P* less than 0.05 was considered statistically significant.

## Results

### Analysis of CYDXF

**Figure 1:**
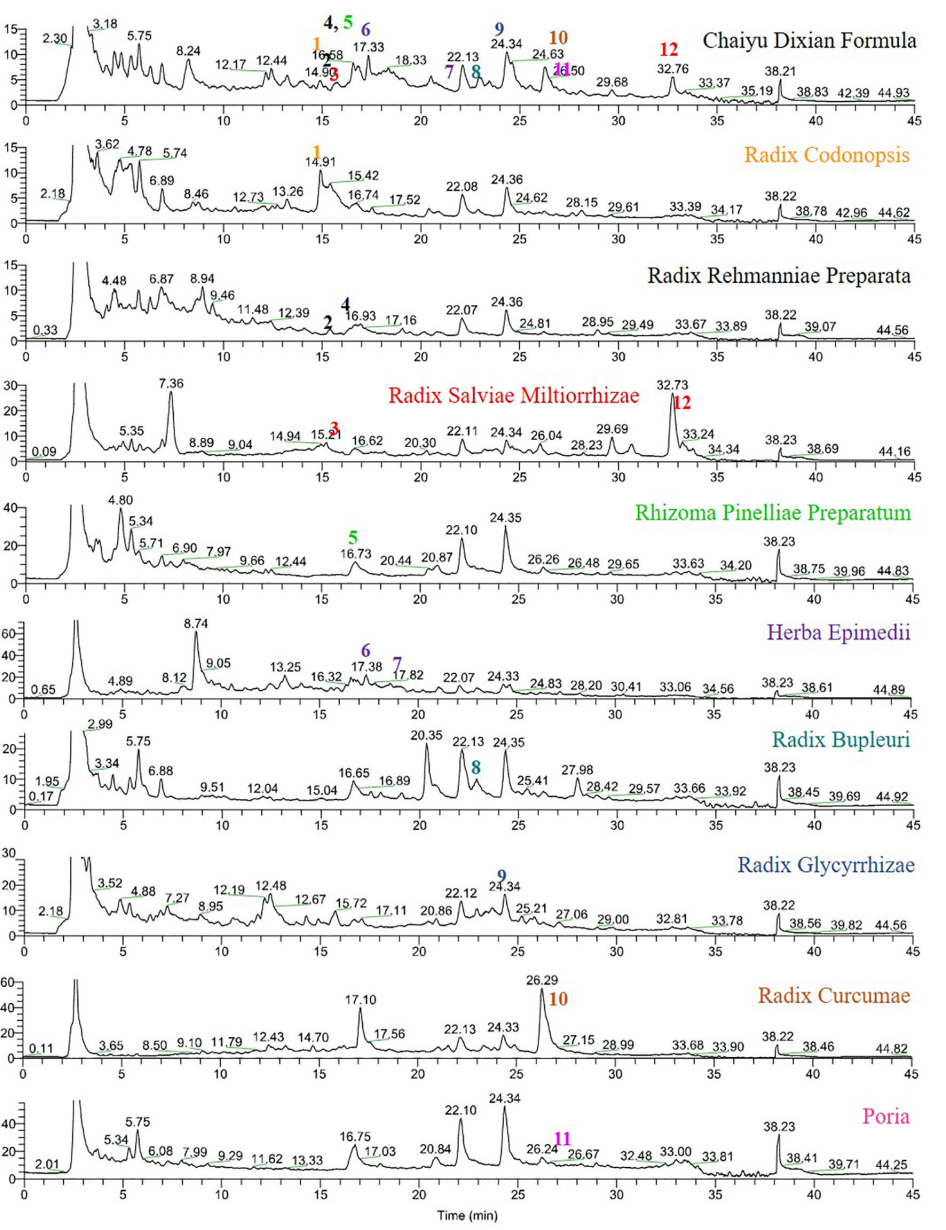
UHPLC-MS fingerprint chromatogram of CYDXF and each herb decoction. Lobetyolin (**1**), martynoside (**2**), salvianolic acid B (**3**), isomartynoside (**4**), chrysophanic acid (**5**), icariin (**6**), epimedin A (**7**), saikosaponin D (**8**), glycyrrhizic acid (**9**), curcumenol (**10**), poricoic acid A (**11**) and cryptotanshinone (**12**).

As shown in **Figure 1**, CYDXF decoction was separated with a UHPLC system and its chromatographic fingerprinting was established (**Figure 1**). Twelve compounds (See **Table 3**) with different retention times were identified as lobetyolin (peak 1), martynoside (peak 2), salvianolic acid B (peak 3), isomartynoside (peak 4), chrysophanic acid (peak 5), icariin (peak 6), epimedin A (peak 7), saikosaponin D (peak 8), glycyrrhizic acid (peak 9), curcumenol (peak 10), poricoic acid A (peak 11), and cryptotanshinone (peak 12) respectively.

**Table 3:** ESI ion peaks elucidated from the UHPLC-MS spectra of the CYDXF and each herb decoction.

Herb	Compound	No.	FW	MW	ESI ion peaks in Herb			ESI ion peaks in Formula		
					tR (min)	ESI^-^	ESI^+^ *	tR (min)	ESI^-^	ESI^+^ *
Radix Codonopsis	lobetyolin	**1**	C_20_H_28_O_8_	396	14.93	–	419.1648	14.88	–	419.1666
Radix Rehmanniae Preparata	martynoside	**2**	C_31_H_40_O_15_	652	15.40	651.2282	653.2282, 675.2239	15.37	651.2295	–
	isomartynoside	**4**	C_31_H_40_O_15_	652	16.27	651.2282	675.2240	16.28	651.2292	675.2254
Radix Salviae Miltiorrhizae	salvianolic acid B	**3**	C_36_H_30_O_16_	718	15.81	717.1477	741.1430	15.64	717.1456	719.1940
	cryptotanshinone	**12**	C_19_H_20_O_3_	296	32.67	295.2270	297.1480	32.49	295.2270	297.1478
Rhizoma Pinelliae Preparatum	chrysophanic acid	**5**	C_15_H_10_O_4_	254	16.29	253.0500	255.0643	16.25	–	255.0642
Herba Epimedii	icariin	**6**	C_33_H_40_O_15_	676	17.32	–	677.2419, 699.2259	17.33	–	677.2436
	epimedin A	**7**	C_39_H_50_O_19_	822	18.99	821.2507	823.2628, 845.2454	21.16	821.2478	823.2610
Radix Bupleuri	saikosaponin D	**8**	C_42_H_68_O_13_	780	22.85	779.4597	781.4700, 803.4494	22.89	779.4583	781.4732, 803.4533
Radix Glycyrrhizae	glycyrrhizic acid	**9**	C_42_H_62_O_16_	823	24.11	821.3935	824.4110	24.21	822.3937	823.4075
Radix Curcumae	curcumenol	**10**	C_15_H_22_O_2_	234	26.27	–	235.1689, 257.1601	26.31	–	235.1691, 257.1504
Poria	poricoic acid A	**11**	C_31_H_46_O_5_	498	26.96	497.3260	–	27.02	497.3269	–

*[M+H]^+^ or [M+Na]^+^.

### CYDXF Attenuated Depressive-like Behaviors Induced by CUMS

#### Body Weight

**Figure 2:**
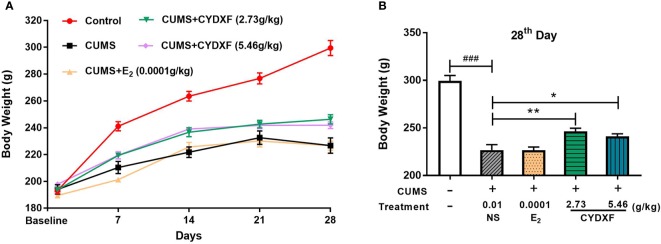
Effect of CYDXF on body weight. **(A)** Changes in body weight during the modeling period; **(B)** The body weight on 28^th^ day. Results are presented as mean ± SEM (n=7). ^###^*P*<0.001 vs control group; *P<0.05 vs CUMS group; **P<0.01 vs CUMS group.

In order to evaluate the effect of CYDXF on the physical condition of CUMS-induced rats, body weight was recorded once a week. As shown in **Figure 2A**, CUMS significantly caused loss of body weight compared to control (*P*<0.001), and both CYDXF (2.73 g/kg) and (5.46 g/kg) treatments significantly alleviated loss of body weight. However, estradiol treatment did not alleviate body weight of CUMS rats. Moreover, on the last day of treatment, both CYDXF (2.73 g/kg) and (5.46 g/kg) did not restore body weight back to that of control but significantly improved body weight compared to no treatment (**Figure 2B**).

#### Behavioral Results

**Figure 3:**
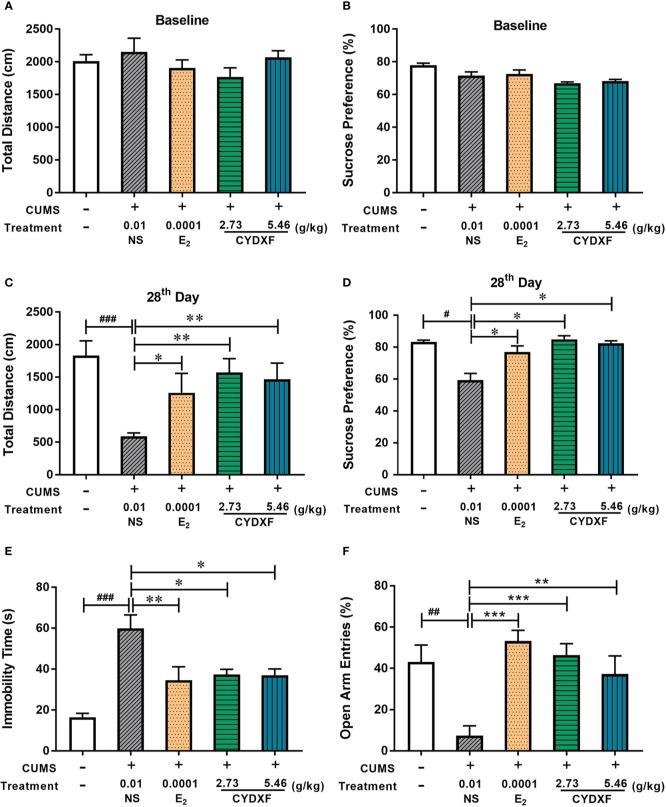
Effect of CYDXF on behavioral tests. **(A)** OFT results on baseline; **(B)** SPT results on baseline; **(C)** The results of total traveled distance on day 28; **(D)** The results of sucrose preference on day 28; **(E)** Immobility time spent in FST; **(F)** Percentage of open arm entries in EPM. Results are presented as mean ± SEM (n=7). ^#^*P*<0.05 vs control group; ^##^*P*<0.01 vs control group; ^###^*P*<0.001 vs control group; *P<0.05 vs CUMS group; **P<0.01 vs CUMS group; ***P<0.001 vs CUMS group.

Several behavioral tests were performed on rats to successfully determine the establishment of the chronic unpredictable mild stress model, and used to evaluate the effect of CYDXF on stressed rats. The OFT was used to examine decreased total traveled distance, which is considered a sign of anxiety-like behavior (Frye et al., 2000). Each group had a similar result of total traveled distance in the OFT at baseline on day 0 (**Figure 3A**, *P*>0.05), and after modeling for 28 days, the total traveled distance of rats in the CUMS group was significantly decreased compared with the control group (*P*<0.001), while estradiol (*P*<0.05) and both CYDXF (*P*<0.01) treatments could significantly alleviate the reduction of total traveled distance (**Figure 3C**). Moreover, the SPT was used to evaluate the motivational state in rodents. Decreased sucrose consumption was a sign of anhedonia (Yu et al., 2011). There were no significant differences at baseline on day 0 among each group, (**Figure 3B**, *P*>0.05), but differences were obvious after the CUMS procedure for 28 days. The sucrose preference was significantly reduced in the CUMS group as compared to the control group (*P*<0.05). Rats exposed to CUMS that were treated with estradiol or CYDXF (2.73 g/kg and 5.46 g/kg) had significantly increased sucrose preference which was restored to a level resembled to that of control group (**Figure 3D**, both *P*<0.05).

Furthermore, The FST and EPM were performed on the final day of the experiment. As shown in **Figure 3E**, rats showed less time spent swimming or struggling but more time spent immobile in the CUMS group compared with the control group (*P*<0.001), indicating that the CUMS caused more depression in rats. However, rats treated with estradiol (*P*<0.01) or CYDXF (both *P*<0.05) significantly reduced immobility time. In the EPM (**Figure 3F**), the percentage of open arm entries in the CUMS rats was significantly lower than the control rats, indicating a high level of anxiety in the CUMS rats. Estradiol- (*P*<0.001) and CYDXF- (2.73 g/kg and 5.46 g/kg) treated rats showed significant improvement in open arm entries as compared to the CUMS group (*P*<0.001; *P*<0.01).

### CYDXF Ameliorated the Abnormality of Rat Estrous Cycle Induced by CUMS

**Figure 4:**
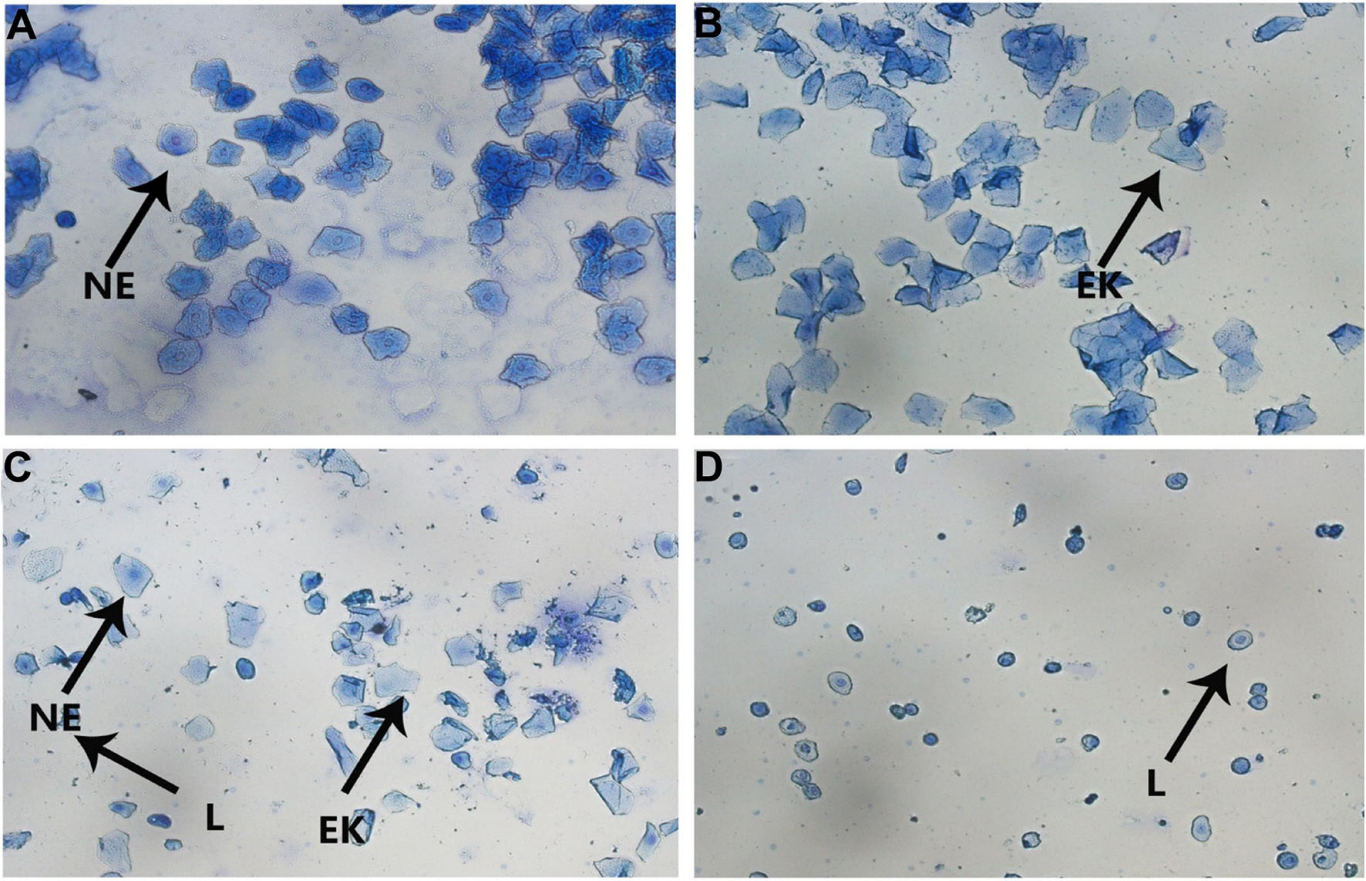
The vaginal smears of rats with different stages of estrous cycle in the control group. **(A)** Proestrus; **(B)** Estrus; **(C)** Metestrus; **(D)** Diestrus (NE, nuclear epithelial cell; EK, epithelial keratinocyte; L, leukocyte).

**Figure 5:**
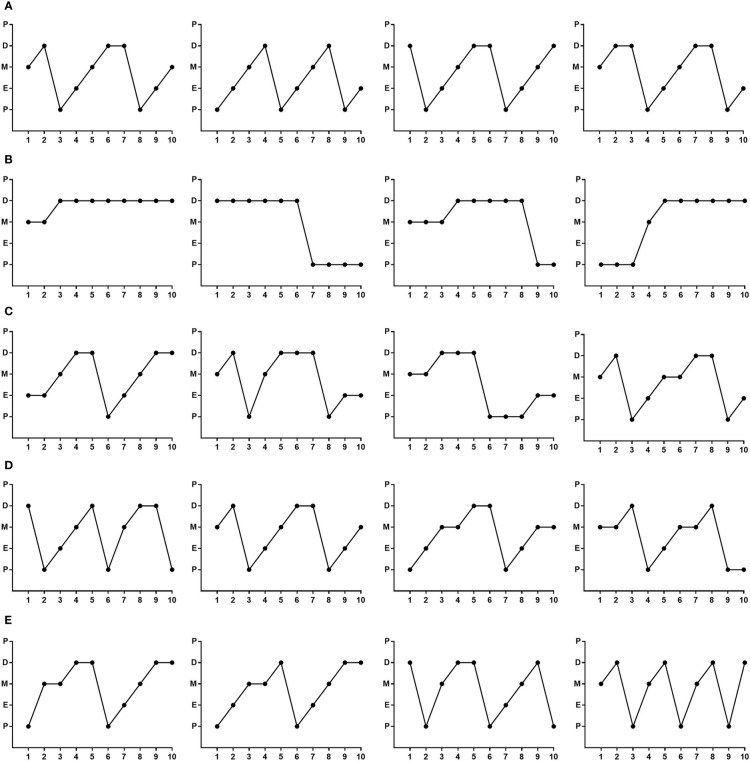
Representative images exhibiting the changes of rat estrous cycle for 10 consecutive days. P, proestrus; E, estrus; M, metestrus; D, diestrus. **(A)** control group; **(B)** CUMS group; **(C)** CUMS+Estradiol group; **(D)** CUMS+CYDXF (2.73 g/kg) group; **(E)** CUMS+CYDXF (5.46 g/kg) group.

The estrous cycle of rats lasts 4-5 days in the control group and is comprised of proestrus, estrus, metestrus, and diestrus, which could be determined according to the cell types through the vaginal smears (**Figure 4**). Rats in the control group (**Figure 5A**) showed normal estrous cycles while those in the CUMS group (**Figure 5B**) experienced irregular estrous cycles. Post-treatments with estradiol (**Figure 5C**), CYDXF (2.73 g/kg) and (5.46 g/kg) revealed that the irregular estrous cycles induced by CUMS were restored (**Figures 5D, E**).

### CYDXF Alleviated Rat Endocrine Disorder Induced by CUMS

**Figure 6:**
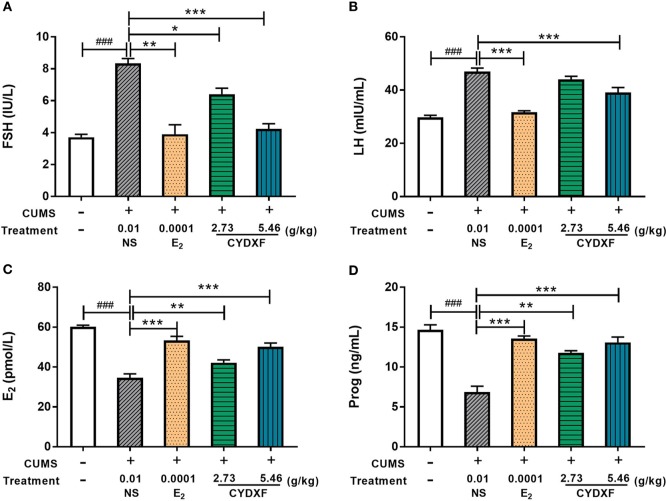
Effect of CYDXF on the concentrations of the hormone levels in serum. **(A)** FSH concentration; **(B)** LH concentration; **(C)** E_2_ concentration; **(D)** Prog concentration. Results are presented as mean ± SEM (n=7). ^###^*P*<0.001 vs control group; *P<0.05 vs CUMS group; **P<0.01 vs CUMS group; ***P<0.001 vs CUMS group.

Daily perceived stress may lead to unknown reproductive disorders (Schliep et al., 2015), which could change the expression of several hormones such as the follicle-stimulating hormone (FSH), luteinizing hormone (LH), progesterone (Prog), and estradiol (E_2_) in serum. As shown in **Figure 6**, rats in the CUMS group showed significantly higher levels of serum FSH and LH (both *P*<0.001) and significantly lower levels of E_2_ and Prog (both *P*<0.001) in comparison to those rats in the control group. Estradiol treatment significantly decreased the serum levels of FSH and LH (*P*<0.01; *P*<0.001, **Figures 6A, B**), and increased the serum levels of E_2_ and Prog of rats in CUMS treatment (both *P*<0.001, **Figures 6C, D**), respectively. Moreover, treatment of CYDXF for rats in CUMS treatment showed significant reversion of these hormones as compared to those of rats in the CUMS group in a dose-dependent manner. Except CYDXF at 2.73 g/kg, which did not significantly alter the level of LH, all other treatments significantly reversed the levels of FSH, LH, E_2,_ and Prog back to the levels of the control group.

### CYDXF Alleviated Rat Follicle Development Abnormality

**Figure 7:**
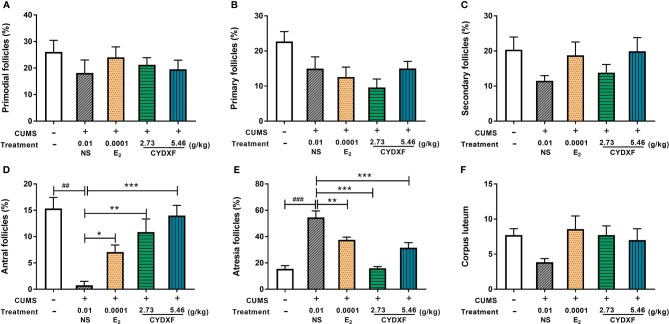
Effect of CYDXF on follicle development. **(A)** Percentage of primordial follicle number; **(B)** Percentage of primary follicle number; **(C)** Percentage of secondary follicle number; **(D)** Percentage of antral follicle number; **(E)** Percentage of atresia follicle number; **(F)** Number of corpus luteum. Results are presented as mean± SEM (n=7). ^##^*P*<0.01 vs control group; ^###^*P*<0.001 vs control group; *P<0.05 vs CUMS group; **P<0.01 vs CUMS group; ***P<0.001 vs CUMS group.

**Figure 8:**
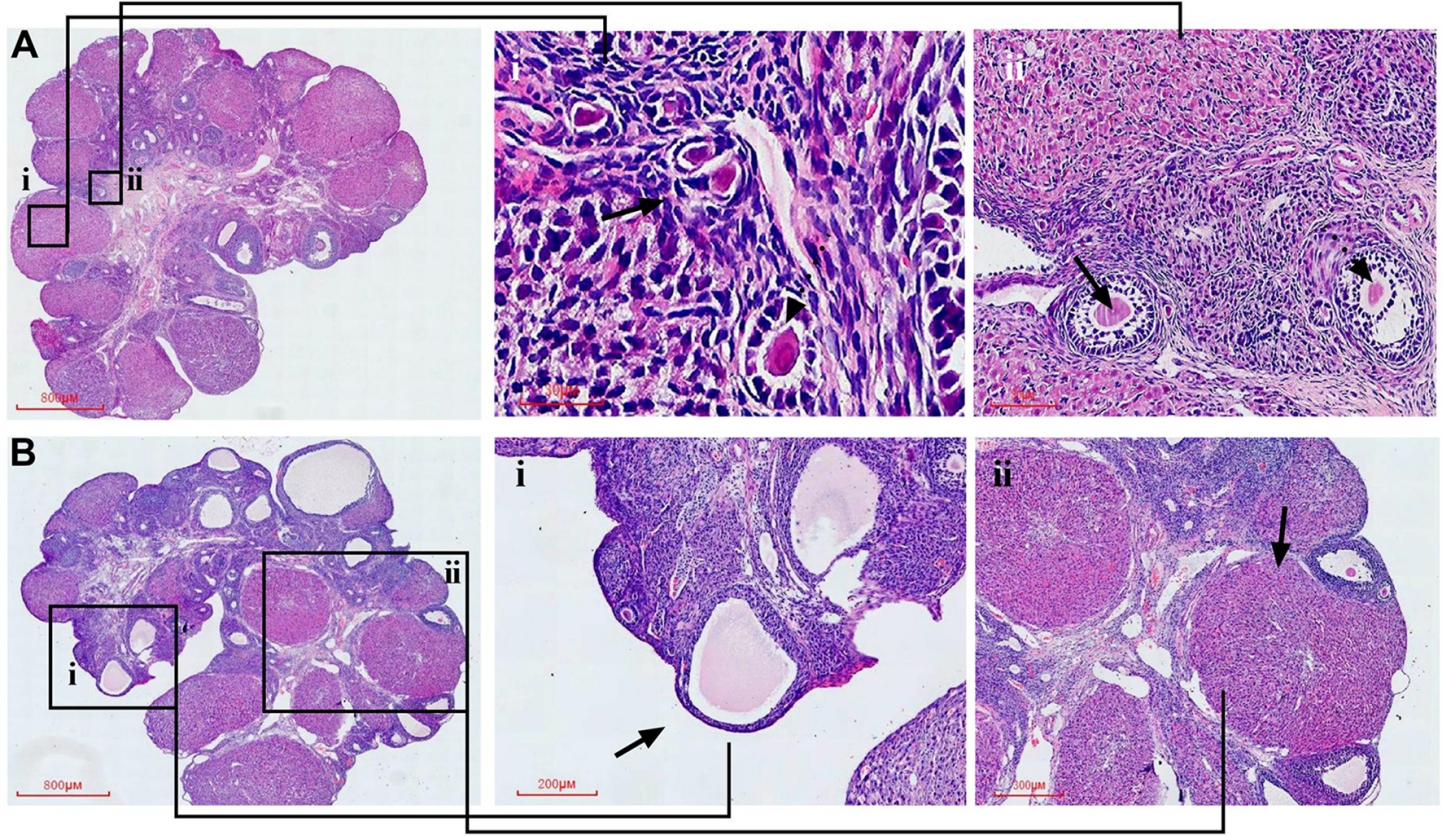
Representative HE staining images of rat ovarian tissues from the control and model group, respectively. Partial enlarged view of the section in **(A)-i** primordial follicle and primary follicle; **(A)-ii** secondary follicle and antral follicle; **(B)-i** atretic follicle; **(B)-ii**, corpus luteum.

Follicle counts were performed on these rats. As showed in **Figures 7** and **8**, representative images of different developmental stages of ovarian follicles as well as the percentage of the follicles and corpus luteum are displayed. In the CUMS group, the percentage of antral follicles was sharply decreased (**Figure 7D**, *P*<0.01), while the percentage of atresia follicles had significantly increased as compared to the control group (**Figure 7E**, *P*<0.001). Treatment with estradiol (*P*<0.05) and CYDXF (2.73 g/kg and 5.46 g/kg) significantly increased the percentage of antral follicles (*P*<0.01, *P*<0.001) (**Figure 7D**). Moreover, both estradiol and CYDXF (2.73 g/kg and 5.46 g/kg) treatments significantly reduced the percentage of atresia follicles (*P*<0.001) (**Figure 7E**). However, there were no significant differences among the percentage of the primordial follicles, the primary follicles, or the secondary follicles, as well as the number of corpus luteum after these treatments (**Figures 7A-C, F**).

### BDNF/PI3K/Akt/mTOR was Involved in CYDXF Treatment of CUMS Rats

In order to examine whether the BDNF-mediated PI3K/AKT signaling pathways were involved in CYDXF-induced ovarian recovery in the CUMS model, the expressions of BDNF/PI3K/Akt/mTOR, as well as the phosphorylation status of Akt and mTOR, were examined. The results indicated that the effects of CYDXF on follicular abnormal development were correlated with the PI3K/AKT signaling pathways activated by BDNF.

**Figure 9:**
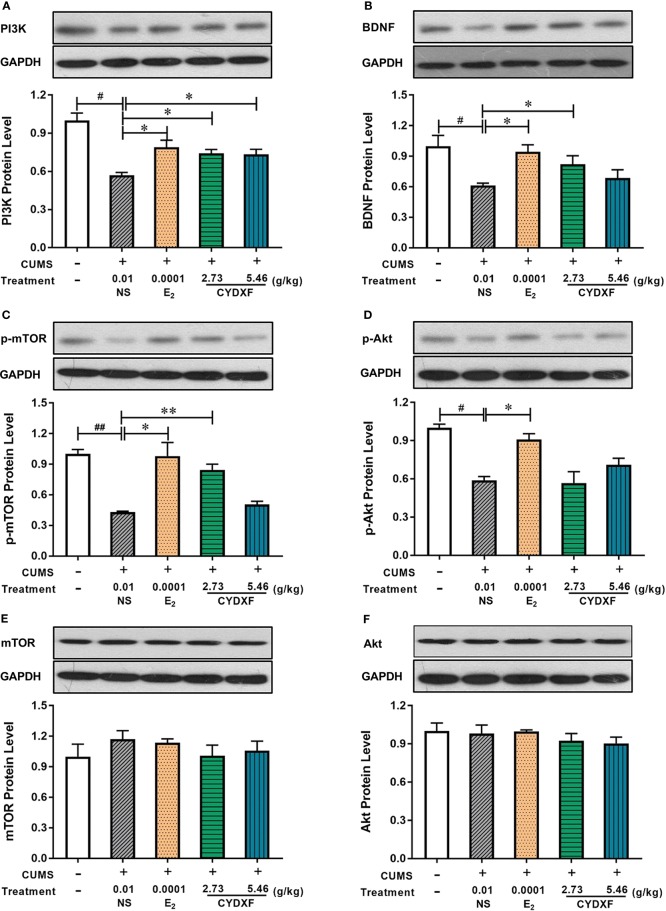
Effect of CYDXF on the protein expression of BDNF mediated PI3K-Akt-mTOR by western blotting. **(A)** PI3K protein expression level; **(B)** BDNF protein expression level; **(C)** p-mTOR (cleaved mTOR) protein expression level; **(D)** p-Akt (cleaved Akt) protein expression level; **(E)** mTOR protein expression level; **(F)** Akt protein expression level. Results are presented as mean ± SEM (n=7). ^#^*P*<0.05 vs control group; ^##^*P*<0.01 vs control group; *P<0.05 vs CUMS group; **P<0.01 vs CUMS group. (The same loading control in **Figure 9C** and **Figure 9E** or **Figure 9D** and **Figure 9F** was used for uncleaved and cleaved versions of the same protein for ease of comparison).

As shown in **Figure 9**, the estradiol and both CYDXF treatments remarkably increased the level of BDNF and PI3K compared with those in CUMS group, respectively (*P*<0.05). Total Akt and mTOR protein expression were almost the same in each group, whereas the p-Akt and p-mTOR levels were markedly lower in the CUMS group than in the control group, respectively (*P*<0.05, *P*<0.01). Compared with those in the CUMS group, estradiol and CYDXF (5.46 g/kg) treatments significantly increased the protein level of p-Akt (*P*<0.01). Meanwhile, as compared to the model group, the relative level of p-mTOR protein was significantly elevated in the estradiol-treated group and CYDXF-treated group at a dosage of 2.73 g/kg, respectively (*P*<0.05). Together, the above results revealed that CYDXF could affect the PI3K/AKT signaling pathways activated by BDNF.

## Discussion

Stress is inevitable in our lives. It is known that stress exerts a detrimental effect on physical and mental status through a healthy person's various systems, including their reproduction system. However, the mechanism of stress that impacts the female reproductive system remains unclear. Many previous studies focused on the effect of stress on the HPA axis. However, in this study, we found that chronic unpredictable mild stress (CUMS) not only induced irregular estrous cycles and endocrine disturbance, but also caused follicle development abnormality, which may be due to the suppression of BDNF-PI3K-Akt-mTOR signaling molecules. CYDXF treatment partly prevented stress-induced downregulation of expression of BNDF/PI3K/Akt/p-mTOR proteins in the ovaries, which was positively correlated with a normalization of hormonal imbalance and most importantly with the stress protective effect of CYDXF on follicular development. The results from this study are in line with those common to stress protecting herbal extracts used to upregulate the PI3K/Akt signalling pathway (Panossian et al., 2018). Follicular maldevelopment could affect female reproduction. Since not all people are suitable for hormone replacement therapy for a long time (Nelson et al., 1997), traditional Chinese medicine showed a great promise in the application for treatment of follicle development abnormality in clinics.

In the current study, we used chronic unpredictable mild stress (CUMS) to induce psychosocial stress in rats. As a putative model of depression, CUMS induced an anxiety-like behavior in the OFT, EPM, as well as a depressive-like behavior in the FST and SPT. Apparently, all the tests above are measuring “behavioral despair” through different methods. In the present study, exposure to stressors caused a decrease in body weight concomitant with a marked change in core behavioral features of depression, including lower motivation, as well as anhedonia (Patki et al., 2013). Taken together, all these indications showed that the rats were experiencing stress and the CUMS model was established successfully.

In TCM theory, follicle development abnormality is based on kidney deficiency and chronic stress can induce liver depression. CYDXF can sooth the liver and nourish the kidney, which is commonly used to treat gynecological diseases and mood disorder. Radix Bupleuri and Radix Curcumae in CYDXF could soothe the liver and relieve depression as well as activate blood circulation and move stagnation. Radix Rehmanniae Praeparata and Epimedium Herb could nourish Yin and Yang together, supplement kidney qi, and promote the viscera function to restore its normal state. Rhizoma Pinellinae Praeparata could dissolve phlegm and dampness as well as strengthen the spleen and stomach. Radix Codonopsis and Poria could fortify the spleen, nourish the heart and calm the mind. The whole formula could invigorate the kidney and soothe the liver, strengthen the spleen and nourish the heart, promote blood circulation and resolve phlegm, which are in line with the basic pathogenesis of chronic stress-induced abnormal ovarian follicular development.

**Figure 10:**
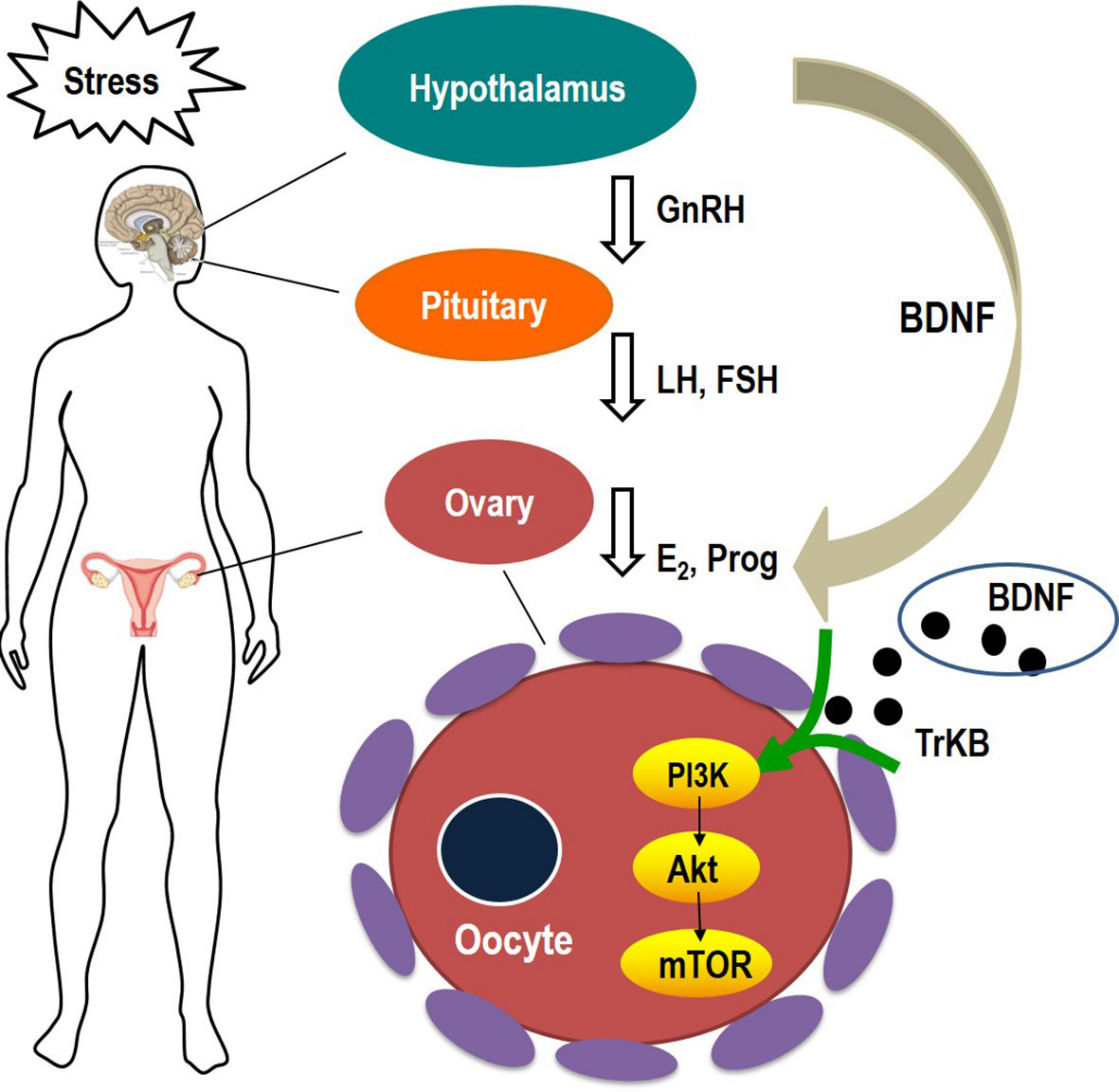
Schematic diagram showing the course of stress inducing follicular abnormal development.

Our findings revealed that CYDXF had multiple functions (**Figure 10**), including producing antidepressant-like effects and treating gynaecological disorders. Firstly, it could alleviate the incidence of CUMS-induced depression-like behaviors. It could not only increase body weight and sucrose consumption in SPT, but also display higher locomotive activity in OFT, more entrance in the open arms in the EPM, and decrease immobility time in FST. The CYDXF contains a variety of components and twelve major bioactive components were found. Both Saikosaponin D from *Bupleurum chinensis* DC. and Curcumenol from *Curcuma aromatica* Salisb. have antidepressant-like activity (Zafir and Banu, 2007; Li et al., 2017). Secondly, CYDXF could regulate the serum hormone levels. In related mechanistic research, stress exerts an impact on the female reproductive system, including the pituitary luteinizing hormone and follicle stimulating hormone, as well as ovarian estrogen and progesterone secretion (Kalantaridou et al., 2004). CYDXF could promote the secretion of estrogen and progesterone and reduce the luteinizing hormone and follicle stimulating hormone, indicating that it may play a role in regulating the hypothalamic-pituitary-ovarian axis to affect female ovulation induction and fertility. We speculate that these actions are probably through the effect of regulating the endocrine system of some components (Zhang et al., 2008). Martynoside, a major component of the *Radix Rehmanniae Praeparata*, has been shown to have antiestrogenic properties (Papoutsi et al., 2006). Specifically, CYDXF could improve the morphology of ovarian tissue. In the present study, exposure to stressors caused marked changes in the antral follicles as there was a significant increase in the atretic follicles. Conversely, CYDXF could promote follicle maturation while decreasing atretic follicles. As one of the major herbs of CYDXF, *Epimedium brevicornu* Maxim can partially restore ovarian function and enhance both fertility (Wang et al., 2019) and the identified component icariin (Ming et al., 2013; Wang et al., 2019). In addition, *Poria cocos* (Schw.) Wolf has antidepressant and immunosuppressive activities (Zhang et al., 2018) and its major component, poricoic acid A, could attenuate oxidative stress and inflammation (Chen et al., 2019). In addition, *Radix codonopsis*, whose main active component is lobetyolin, could improve the cognitive-function and neuroprotective effects (Weon et al., 2013). *Salvia miltiorrhiza* Bunge exerts estrogenic effects on reproductive tissues (Xu et al., 2016). Its two major bioactive compounds, Salvianolic acid B and cryptotanshinone, were reported to have an antidepressant-like effect (Zhang et al., 2016) and reverse reproductive and metabolic disturbances through regulating ovarian signaling pathway (Yang et al., 2011), respectively. Glycyrrhizic acid, a bioactive compound of *Glycyrrhiza uralensis* Fisch., also has a therapeutic effect on neural disease by influencing the PI3K/Akt signaling pathway (Kao et al., 2009). Furthermore, chrysophanic acid has been proven to suppress activation of signaling molecules, such as AKT and mTOR (Lee et al., 2011).

One of the major findings in our study was the involvement of the BDNF-mediated PI3K/Akt/mTOR signaling pathway. We found that the phosphorylation of Akt and mTOR were significantly decreased in CUMS rats. By contrast, CYDXF treatment could obviously increase the phosphorylation of Akt and mTOR. In addition, CUMS caused a reduction of PI3K and BDNF expression in rats, and these changes were attenuated by CYDXF. BDNF was originally discovered in the nervous system as a stress responsive messenger (Larsen et al., 2010; Panossian et al., 2018), but has recently shown to be related to human reproductive diseases including polycystic ovary syndrome, infertility, and impaired ovarian oocyte development (Wu et al., 2012a; Russo et al., 2012; Sadeu et al., 2012). The phosphoinositide-3 kinase (PI3K) signaling pathway plays an essential role in oogenesis, folliculogenesis, as well as ovulation in the ovary (Zheng et al., 2012). Although the basic mechanism of the PI3K/Akt/mTOR signaling pathway has been well studied, much remains to be learned when it comes to reproductive biology. The PI3K pathway, which is characterized by regulating cell survival, growth and proliferation, may also play a critical part in the regulation of oocyte growth and the activation of primordial follicles (Liu et al., 2006). PI3K is also activated by FSH and LH, which are essential hormones for ovulation (Dupont et al., 2012). The protein kinase B (Akt), which cooperates with other kinases to regulate follicular development, determines the pool of primordial follicles which transform from a quiescent to a growing phase in the mammalian ovary (Cecconi et al., 2013). The mammalian target of rapamycin (mTOR) is an important downstream of PI3K/Akt signaling. Recent study has shown that mTOR signaling cascade suppresses granulosa cell autophagy (Choi et al., 2013), and abnormalities in this process may cause reproductive diseases such as premature ovarian failure and polycystic ovary syndrome (Adhikari and Liu, 2010; Yaba and Demir, 2012).

From our study and literature, we speculated that the main reason for the efficacy of CYDXF was probably due to the activation of AKT, while some of the active ingredients were responsible for this effect. Therefore, the restoration of BDNF/PI3K/Akt/mTOR expression by CYDXF may be attributed to, at least in part, the effects of regulating endocrine disorders and/or treating the depressive disorders of these bioactive compounds.

In a word, our study first manifested that CYDXF has multiple functions, including improvement of depression-like behaviors, but is not limited to the regulation of serum hormone levels and promotion of the ovarian function. However, several limitations in our study still exist. First of all, due to the great complexity in Chinese medical formula, it remains unknown which herb or component in CYDXF is mainly responsible for the beneficial effects, how they exert their signaling functions requires further investigation. Secondly, whether CYDXF is effective in other models of depression remains to be further studied. In addition, clinical observations of its randomized trials in humans are needed. The present study which builds the relationship between stress and reproduction is the first step towards this investigation.

## Conclusion

Chaiyu-Dixian Formula could attenuate chronic stress-induced abnormal ovarian follicular development through relieving depression-like behaviors and ovarian function. Its mechanism was partly attributed to the regulation of BDNF-mediated PI3K/Akt pathway.

## Data Availability Statement

All datasets generated for this study are included in the article/supplementary material.

## Ethics Statement

The animal study was reviewed and approved by the Laboratory Animal Administration and Ethics Committee of Xiamen University (Permission license No. XMULAC 20190020).

## Author Contributions

H-XX carried out the experiment and contributed to the compilation of data and the writing of this paper. YG critically revised the manuscript. H-MZ and S-YX contributed to data analysis and administrative support. S-XL, Z-XH, and C-YZ participated in the experimental process and contributed to the collection of data.

## Funding

This work was supported by the Natural Science Foundation of Fujian Province of China (No. 2017J01147), the Traditional Chinese Medicine Foundation of Fujian Ministry of Health of China (No. wzpw201307), the Natural Science Foundation of Fujian Province of China (No. 2018J01136) and the National Natural Science Foundation of China (No. 81202659).

## Conflict of Interest

The authors declare that the research was conducted in the absence of any commercial or financial relationships that could be construed as a potential conflict of interest.

## Abbreviations

CYDXF, Chaiyu-Dixian Formula; CUMS, Chronic unpredictable mild stress; NS, Normal saline; ELISA, Enzyme-linked immunosorbent assay; BDNF, Brain-derived neurotrophic factor; PI3K, Phosphoinositide-3 kinase; Akt, Protein kinase B; mTOR, Mammalian target of rapamycin; NE, Nuclear epithelial cells; EK, Epithelial keratinocyte; L, Leukocyte; OFT, Open filed test; FST, Forced swim test; SPT, Sucrose preference test; EPM, Elevated plus maze; FSH, Follicle-stimulating hormone; E_2_, Estradiol; LH, Luteinizing hormone; Prog, Progesterone.
